# Endocrine disrupting chemicals (EDCs) and female cancer: Informing the patients

**DOI:** 10.1007/s11154-016-9332-9

**Published:** 2016-02-01

**Authors:** Dominik Rachoń

**Affiliations:** Department of Clinical and Experimental Endocrinology, Medical University of Gdańsk, Dębinki 7, 80-211 Gdańsk, Poland

**Keywords:** Endocrine disrupting chemicals (EDCs), Female cancer, Breast cancer, Uterine cancer, Ovarian cancer, Cervical cancer

## Abstract

Breast and uterine cancer are the most frequent female gender related neoplasms whose growth is mostly estrogen dependent. Therefore, any EDC exhibiting estrogenic effects may increase the risk of these two malignancies. This review focuses on the potential role of EDCs with estrogenic potential on the risk of breast and uterine neoplasms but also points to the possible role of the exposure to EDCs in the pathogenesis of ovarian and cervical cancer. It also underlines the necessity of informing the public about the presence of EDCs in common consumer products, their detrimental health effects and methods of reducing the exposure risk.

## Introduction

Endocrine disrupting chemicals (EDCs) are environmental compounds (natural or synthetic), which impair the function of the endocrine system leading to adverse health outcomes. A group of chemicals with an endocrine disrupting potential is very heterogeneous and includes many synthetic substances used in agriculture, industry as well as many consumer products. The most common include polychlorinated biphenyls (PCBs), polybrominated diethyl ethers (PBDEs), dioxins, plasticizers [bisphenol A (BPA) and phthalates], pesticides [methoxychlor, chlorpyrifos, dichlorodiphenyltrichloroethane (DDT)], fungicides (vinclozolin) and herbicides [1, 2]. Also several naturally occurring plant derived substances possess an endocrine disrupting potential (phytoestrogens) [3, 4]. Although there are several different mechanisms through which EDCs can impair the function of the endocrine system [2, 5], most of the reported adverse effects of their action are due to their interaction with estrogen receptors (ERs) or alteration of estrogen signaling pathways [6].

Female gender related neoplasms include breast, uterine, ovarian and cervical cancer. The first two are estrogen dependent malignancies, therefore their development and growth is usually due to the prolonged or exaggerated exposure to estrogens [7]. It is still debatable if ovarian cancer is estrogen dependent, whereas cervical cancer is mostly due to infection with oncogenic viruses [the human papilloma virus (HPV)] [8].

### BPA as the most abundant EDC exhibiting estrogenic properties

BPA was first developed in the 1890s as a synthetic estrogen which in the 1930s has been shown to possess estrogenic activity comparable to estrone in stimulating the female reproductive system in rats [9]. Surprisingly, data on the estrogenic properties of this compound have been forgotten and nowadays BPA is the most abundant chemical used in many consumer products [10]. It is mostly used in the production of polycarbonate plastics and epoxy resins, food packaging, dental sealants, and thermal receipts. Therefore, BPA can migrate into dust from laminate flooring, adhesives containing epoxy resins, paints, toys and household electronic equipment [11]. BPA is also widely used as the inside coating of cans used for food preservation and storage. Heating cans during sterilization or food preparation causes BPA to leak into the can content and therefore, BPA is also found in many food products [12]. Consequently, we are now exposed to this chemical not only via inhalation of household dust but mostly by eating foods stored in plastic containers or cans [10]. Due to the phenolic structure, BPA has been shown to interact with estrogen receptors (ERs) and estrogen signaling pathways [13]. The binding affinity of BPA with both ERs has been estimated to be 0.1–0.01 % of the affinity of 17β-estradiol (E2) [14] but data from the *in vitro* experiments on ER positive human MCF-7 breast cancer cells show that although it competes more effectively for binding with the ERα it induces the ERα- and ERβ-mediated gene expression with comparable efficacy [15]. BPA has also been shown to activate the expression of the target genes by signaling via so-called membrane estrogen receptors [G-protein coupled ERs (GPER)] with the potency comparable to the E2 or through related signal-transduction cascades [16]. Although BPA is generally considered to be an ER agonist in some tissues it has been shown to have antagonistic effects (i.e., brain and uterus) [17]. In addition to binding to ERs, BPA has also been shown to have an influence on their expression. Data from *in vitro* as well as *in vivo* experiments showed that exposure to this phenolic compound leads to the upregulation of ERα gene expression in different cell types and tissues [18–21]. Apart from the apparent affinity of this chemical to the ERs, BPA has also been shown to bind androgen receptors (AR) [22, 23], thyroid hormone receptors (TR) [24] and peroxisome proliferator activated receptors gamma (PPARγ) [25]. Therefore, BPA may not only cause adverse effects in the reproductive tissues but may also greatly influence metabolic aspects of human health (reviewed in ref. [26, 27]).

### Carcinogenesis of estrogen dependent female cancer

Breast and uterine cancer are the most frequent female gender related neoplasms whose growth is mostly estrogen dependent [7]. Therefore, any EDC exhibiting estrogenic effects may increase the risk of these two estrogen dependent malignancies. The results of a vast number of experiments conducted in rodents stress the importance of fetal as well as pre-pubertal exposure to EDCs in the pathogenesis of breast and uterine cancer [28, 29]. Altering the hormonal milieu *in utero* as well as before puberty can have detrimental effects on breast and uterine morphology and function in adult life (reviewed in ref. [30]). Carcinogenesis is a process, which is usually looked upon as a consequence of DNA mutations in the genes that control cell proliferation, differentiation and maturation – the so called somatic mutation theory (SMT) [31]. However, recent data from several studies point to the role of cell to cell communication and cell-matrix interactions which when gone awry result in disruption at the tissue-level that give rise to malignancy – the so called tissue organization field theory (TOFT) [32]. Breast tissue morphogenesis involves many reciprocal interactions between the stroma and the epithelium, thus the TOFT is more likely to explain the pathogenesis of breast cancer [33]. Fetal expression of the two ER isoforms (ERα and ERβ) is exclusively detected in the stroma of developing mammary gland [34, 35] which points to the role of this tissue compartment in proper breast development. Therefore, any imbalance in the exposure to hormonal and growth factors, which govern the proper development of the stromal and epithelial tissue of the developing mammary gland may lead to the formation of neoplasia later in adulthood. Experimental data from developing breast tissue in mice and rats, clearly point to such a relationship [33].

### Direct effects of EDCs on the development of estrogen dependent cancer

Mammary gland and the uterus are the main estrogen target organs therefore studies on the carcinogenic effects of EDCs mostly concentrate on those exhibiting estrogenic potential. Most data stem from the studies on BPA since it is the most prevalent estrogenic EDC found in many consumer products [10, 36, 37]. Growth promoting effects of BPA in different cell lines expressing ERs (i.e., MCF-7) only proof its estrogenic properties and do not necessary point to the carcinogenic potential of this compound [37]. However, data from a great number of studies conducted on animal models of breast cancer show that BPA may actually promote the growth of this malignancy also via non-estrogen dependent pathways (reviewed in ref. [37, 38]). It has been also stressed that the time of exposure to this compound has a great impact on breast cancer risk [39]. Interactions with the peri-ductal stromal breast tissue during fetal mammary gland development seem to have a tremendous impact on the development of this malignancy (reviewed in ref. [40]). Clinical data on the risk of breast cancer development due to the exposure to estrogenic EDCs are limited and inconclusive [41–44]. Recently however in a very interesting and a unique case–control study conducted by Cohn et al. [45] it has been found that *in utero* exposure to the estrogenic pesticide DDT is associated with an increased risk of breast cancer later in life.

Surprisingly, data on the role of EDCs in the pathogenesis of uterine hyperplasia and cancer is very limited. The first study evaluating the effects of EDCs on endometrial morphology in women was conducted by Hiroi et al. [46]. These authors demonstrated a possible association between the exposure to BPA and estrogen dependent endometrial disorders. However, no association between endometrial cancer and PCB congeners, DDT-related and organochlorine compounds was found in a study conducted by Sturgeon et al. [47]. Surprisingly, the results from the studies on dietary isoflavones show a decreased risk of endometrial cancer in women [48, 49], whereas data from animal experiments point to the uterotrophic potential of plant derived substances and their estrogenic metabolites. For example equol – a metabolite of daidzein present in soy, has been shown to cause endometrial growth and hyperplasia in ovariectomized rats [50]. These results were consistent with a study of Unfer et al. [51] where women taking soy extracts to alleviate menopausal symptoms had a higher incidence of endometrial hyperplasia compared to those who were taking placebo. Therefore, studies on the association between the exposure to estrogenic EDCs and the risk on endometrial hyperplasia and cancer are still warranted.

### Indirect effects of EDCs on the risk of estrogen dependent cancer

Apart form the direct actions of estrogenic EDCs on the estrogen target organs, they may also indirectly affect the risk of cancer development by influencing other risk factors. For instance, early menarche and late menopause prolong women’s lifetime exposure to estrogens thus increasing also the risk of estrogen dependent malignancies [52]. Therefore, any EDCs having an influence on these two events in women’s life, may also significantly impact the risk of breast and uterine cancer development [7, 53]. Data from studies conducted in humans linking the exposure to EDCs and age at menarche are very limited and inconclusive. In a study conducted by Vasiliu et al. [54] *in utero* exposure to dichlorophenyldichloroethylene (DDE) reduced age at menarche by 1 year. However, when controlling for estimated body size at menarche this association was no longer significant. Similarly, cross-sectional data from the National Health and Nutrition Examination Survey (NHANES) showed that adolescent girls with moderate urine BPA concentrations appeared to be less likely to have early onset of menarche then those with the lowest. Yet, while the magnitude of the studied association remained unchanged when likely confounders were taken into the consideration, the results were no longer statistically significant. Additionally, overweight status strongly modified the association between the urinary BPA concentrations and the age of menarche [55]. Clinical data on the influence of exposure to EDCs and the timing of menopause are still lacking.

EDCs that interact with ERs or estrogen signaling pathways may also indirectly influence the risk of estrogen dependent cancer via the action on the hypothalamic-pituitary-gonadal axis. EDCs exhibiting anti-estrogenic actions in the hypothalamus increase the secretion of gonadotropins and thus the production of the ovarian steroids, which in turn increases the exposure to unopposed high estrogen concentrations. Paradoxically, EDCs exhibiting estrogenic actions may also lead to the sustained elevation of LH (“positive” feed-back loop), which leads to an exaggerated androgen production in the theca cells - a common feature observed in women with PCOS [56]. Data from several clinical studies are consistent with this hypothesis. Women with PCOS develop unovulatory infertility and also have a higher risk of uterine cancer [57]. Clinical data on the risk of breast cancer risk in these patients are however still inconsistent [58].

Obesity is another factor that has a great impact on breast and uterine cancer risk [59, 60]. This association is due to the aromatase overexpression in dysfunctional adipose tissue which converts androstenedione of adrenal origin into estrone which can be further converted into E2 by the activity of 17β-hydroxysteroid dehydrogenase, leading to unopposed hyperestrogenemia [61]. Therefore, factors that cause or induce obesity can also have a great impact on the breast and uterine cancer risk, especially in postmenopausal women. Data from recent studies proof that prenatal exposure to EDCs is associated with the development of obesity later in life [62]. Also it has been shown that dietary ingestion of persistent organic pollutants (POPs) promote the development of obesity, which in turn influences breast cancer risk [63]. Data from studies on the metabolic effects of BPA also point to the obesogenic potential of this EDC (reviewed in ref. [64]).

Breast tissue is also a target organ for the action of prolactin (PRL), which is responsible for the initiation of lactation postpartum. The production of PRL by the pituitary lactotrophic cells is mostly via the action of estrogens and their signaling pathway. Therefore, any EDC exhibiting estrogenic activity may stimulate PRL secretion. This has been actually proven in animal experiments [4, 65]. Whereas exposure to high PRL levels is a risk factor for breast cancer development is still debatable [66]. Nevertheless, such an association is justifiable since the breast tissue is the main target organ for the PRL action where it stimulates its growth and maturation not only postpartum but also during puberty [67]. Clinical data on the effects of human exposure to EDCs on PRL secretion are, however, still lacking.

### Ovarian cancer and EDCs

Although ovarian cancer is not an estrogen dependent malignancy [68] there have been some studies conducted *in vitro* on the effects of selected EDC on the proliferation of ovarian cancer cells. Data from experiments conducted *in vitro* showed that BPA, nonylphenol, octylphenol, methoxychlor, benzophenone-1 stimulated the proliferation of ER positive BG-1 ovarian cancer cells via estrogen signaling pathway [69, 70]. Benzophenone-1 is a commonly used UV-filter, which has also been shown to possess immunomodulating properties, which resemble those of E2 [71]. Data from the experiments conducted by Hall and Korach [72] also pointed to the mitogenic potential of BPA, genistein and 2,2-bis(p-hydroxyphenyl)-1,1,1-trichloroethane (HPTE) in ovarian cancer cells, which was regulated via ER-mediated expression of the chemokine CXCL12 (stromal cell-derived factor-1). The results of these studies, however, do not point to the potential association between exposure to these substances and ovarian cancer risk. We might speculate on the potential role of the indirect actions of EDCs on ovarian cancer risk, especially those possessing anti-estrogenic effects in the hypothalamus leading to an increased production of gonadotropins, which directly stimulate the gonads and therefore may also impact the risk of ovarian cancer development [73]. Clinical data on the exposure to environmental EDCs and the risk of ovarian cancer is, however, still lacking.

### EDCs and cervical cancer

Although cervical cancer is also not an estrogen dependent neoplasm the first data on the deleterious effects of exposure to EDCs actually come from the observations that women exposed *in utero* to diethylstilbestrol (a synthetic estrogen which was prescribed until 1971 to pregnant women to prevent miscarriages) developed vaginal clear cell adenocarcinoma [74]. These observations have stressed the role of time of exposure to EDC during the stages of fetal development and its consequences later in adult life. Nowadays, infection with human papilloma virus is the main culprit of cervical cancer [8]. Nevertheless, a recent study conducted *in vitro* by Ma et al. [75] showed that nanomolar concentrations of BPA promoted migration and invasion of cervical cancer HeLa, SiHa, and C-33A cells. Further studies on the potential role of exposure to other estrogenic EDCs on the risk of cervical cancer are however still warranted.

### Informing the patients on EDCs and the risk of female gender related cancer

It is now apparent that EDCs that interact with ERs or estrogen signaling pathways may have detrimental effects on women’s reproductive health. A multidisciplinary approach is needed to reduce the exposure to these chemicals where not only gynecologists [76] and endocrinologists, but mostly family doctors should be involved in the process of widening the knowledge and the awareness about the consequences of exposure to these environmental substances [77]. Although, there is vast information available from the governmental (http://edkb.fda.gov, http://www.epa.gov/endo) as well as private organizations (http://www.ourstolenfuture.org, http://www.silentspring.org, http://www.nrdc.org, http://www.healthandenvironment.org) on the detrimental health effects due to exposure to EDCs, recommendations in an easily accessible format on methods of minimizing the exposure to these substances are still lacking [30].

### Summary

Although more clinical studies are warranted on the association between exposure to EDCs and the development of female gender related malignancies, it is already clear that both the level and timing of exposure are crucial. Also, apart form the direct actions of estrogenic EDCs on the estrogen target organs, they may also indirectly affect the risk of cancer development by influencing other risk factors. Therefore, in order to reduce the risk of female gender related neoplasms, the public should be informed about the presence of EDCs in common consumer products and methods of limiting exposure to these substances.
